# Effects of 8-Week Whole-Body Vibration Training on the HbA1c, Quality of Life, Physical Fitness, Body Composition and Foot Health Status in People with T2DM: A Double-Blinded Randomized Controlled Trial

**DOI:** 10.3390/ijerph17041317

**Published:** 2020-02-18

**Authors:** Francisco Javier Domínguez-Muñoz, Santos Villafaina, Miguel A. García-Gordillo, Miguel Ángel Hernández-Mocholi, Daniel Collado-Mateo, José C. Adsuar, Narcis Gusi

**Affiliations:** 1 Physical Activity and Quality of Life Research Group (AFYCAV), Faculty of Sport Science, University of Extremadura, 10003 Cáceres, Spain; fjdominguez@unex.es (F.J.D.-M.); mhmocholi@unex.es (M.Á.H.-M.); ngusi@unex.es (N.G.); 2 Facultad de Administración y Negocios, Universidad Autónoma de Chile, sede Talca 3467987, Chile; 3 Centre for Sport Studies, Rey Juan Carlos University, Fuenlabrada, 28943 Madrid, Spain; danicolladom@gmail.com; 4 Health Economy Motricity and Education (HEME), Faculty of Sport Science, University of Extremadura, 10003 Cáceres, Spain; jadssal@unex.es

**Keywords:** type II diabetes mellitus, WBV, HbA1c, lipid profile, fasting blood glucose, TUG, fat mass

## Abstract

The aim of this study was to analyze the effects of an 8-week whole-body vibration (WBV) on the quality of life, physical fitness, body composition, glycosylate hemoglobin (HbA1c), lipid profile, and foot health status in people with type II diabetes mellitus (T2DM). It was performed as a double-blinded randomized controlled trial of 90 people with T2DM. Primary care facilities were used. The 8-week WBV training consisted of maintaining a knee flexion at 45° during five to nine series of 30–60 s in a vibration frequency that oscillated between 12.5–18.5 and 30 s of recovery between series. The placebo group had to perform the same protocol but without vibration. Participants performed the protocol three times per week. The WBV training significantly reduced the fat mass (%) of people with T2DM. However, significant effects of WBV training were not found in the quality of life, physical fitness, foot health status, lipid profile, blood pressure, fasting blood glucose, or HbA1c. Nevertheless, within groups enhances were found in HbA1c, blood pressure, fasting blood glucose, foot health status, health-related quality of life, timed-up and go test, and chair-stand test in both WBV and placebo groups. WBV was shown to be beneficial for reducing the fat mass and lipid profile of people with T2DM. The improvements of the placebo group could be due to both the social benefits of enrolling in an intervention and the physical fitness benefits of isometric contractions. Further studies are needed to clarify the effects of WBV and to establish a dose–response relationship in people with T2DM.

## 1. Introduction

Diabetes Mellitus (DM) is defined as a state of hyperglycemia, in either fasting or postprandial states (1). DM is a group of metabolic diseases characterized by hyperglycemia resulting from defects in insulin secretion, insulin action, or both [1,2]. Type 2 diabetes mellitus (T2DM) is one of these diseases and it is characterized by insulin resistance [3]. The International Diabetes Federation (IDF) estimates that the global prevalence of diabetes mellitus is 366 million in 2011 and expects an increase to 552 million in 2030 [4]. In this sense, the increase in the DM prevalence, especially in T2D, has become one of the major problems in modern society [5]. In this regard, this chronic disease is connected with the increase of obesity prevalence [6] and the high levels of physical inactivity [5] in people with T2DM. Peripheral neuropathy is one of the most common complications and consequences of chronic hyperglycaemia [7]. This is characterized by a progressive degeneration that primarily affects the small-diameter cutaneous nociceptive fibres [8].

Physical exercise has become a therapy for people with T2DM [9], improving physical fitness [10,11], quality of life [12], and autonomic modulation [13]; as well as enhancing metabolic control [14] and insulin sensitivity; and reducing inflammatory markers [15] and neuropathy symptoms [16]. Interestingly, physical activity could also increase the regenerative capacity of cutaneous axons, slowing or preventing neuropathy progression [17].

Exercises based on whole-body vibration (WBV) training have shown to enhance balance, mobility muscle strength, pain, cardiorespiratory fitness, and bone density, in both healthy and clinical populations [18,19,20,21,22,23]. In this regard, a recent systematic review and meta-analysis (with a total of seven studies, involving 279 older adults with T2DM) focused on the effects of WBV determined that this training improved the mobility, balance and aerobic capacity without any known adverse effects [24]. Moreover, WBV could have positive effects on glycaemic indices or body composition. Regarding glycaemic indices, a previous study showed that acute WBV decreases the glucose level in elderly women with T2DM. However, the effects of WBV intervention on glycosylated haemoglobin (HbA1c) is uncertain. Whereas some studies have reported significant effects on HbA1c [25,26], others have not observed changes in this relevant variable [27,28]. Additionally, WBV seems to be effective to reduce the body fat mass in people with T2DM [29]. Moreover, a systematic review showed that WBV produces a significant body weight drop, which is quite relevant due to the associated obesity in T2DM [30].

In addition, Gomes-Neto, de Sa-Caputo, Paineiras-Domingos, Brandao, Neves, Marin, Sanudo and Bernardo-Filho [24] stated that WBV could be a useful strategy for the management of T2DM.

However, Gomes-Neto, de Sa-Caputo, Paineiras-Domingos, Brandao, Neves, Marin, Sanudo and Bernardo-Filho [24] stated that further studies are needed to reinforce the hypothesis as well as to determine the optimum dosages and durations of effect. Therefore, the aim of the present study was to investigate the effects of 8 weeks of WBV training on the body composition, lipid profile, glycosylated haemoglobin (HbA1c), fasting blood glucose, physical fitness, and health-related quality of life.

## 2. Materials and Methods

### 2.1. Trial Design

This study was conceived as a double-blinded, randomized controlled trial where participants were randomly allocated into two groups: whole-body vibration (WBV) and placebo group (PG). All the procedures were approved by the University research ethics committee (approval number: 44/2012). The trial was prospectively registered at the International Standard Randomized Controlled Trial Number Registry (ISRCTN16866781).

### 2.2. Participants

The intervention was carried out in the Primary Care Center “Manuel Encinas” from the city of Caceres, in Extremadura, Spain from March 2013 to June 2013.

The HbA1c was used to estimate the sample size. Accepting an alpha risk of 0.05 and a beta risk of 0.05 in a bilateral contrast, at least 40 participants in the WBV group and 40 in the PG are required to detect a difference equal or greater than 0.57 units [31]. Taking into account a previous study [32], we assumed that the common standard deviation was 1 and the correlation coefficient between the pre and post mean was 0.8. Moreover, a follow-up loss rate of 20% has been estimated.

Finally, 90 T2DM patients were included in this randomized controlled trial. All of them were recruited until 31 January, 2013. Participants inclusion criteria were:

(a) Men or women with T2DM diagnosed between 40 and 85 years old. T2DM is characterized by hyperglycemia caused by insulin resistance [3].
(b) Have read, accepted and signed the written informed consent.

Moreover, participants were excluded if they:

(a) have Type I diabetes mellitus diagnose, since the aetiology completely differs from T2DM.
(b) have a condition that may make the high intensity exercises contraindicated, such as retinopathy, musculoskeletal injuries, major balance problems or high risk of thrombosis.
(c) Be under psychotropic or neurotoxic treatment.
(d) Be exposed to neurotoxins (industrial accidents or be in contact with toxic residues).
(e) Receive radiation therapy.
(f) High risk of non-diabetic neuropathy (such as HIV, alcoholism or uraemia).
(g) Have or had a job with high exposure to mechanical whole-body vibrations.
(h) Have performed whole-body vibration exercises prior to this intervention.

Participants were randomly allocated into the two groups (WBV or PG) by a technician using random numbers. Participants allocated to WBV and PG were classified as A and B respectively. This researcher did not take part in the acquisition, intervention or data analysis. A technician who was blinded to the grouping allocation developed the intervention and had to apply protocol A or B for each participant. The placebo group was led to believe that they were receiving a WBV below the vibration threshold that it was able to detect. For this purpose, the Galileo Fitness platform was connected through wiring to a screen where the participant had the values of vibration duration and frequency amplitude, but it really was a software that controlled two speakers placed inside the vibration platform.

### 2.3. Intervention

The procedures conducted by WBV and PG during the 8-weeks training are described below. Whole-body vibration group protocol: Table 1 shows the number of series, time, vibration frequency and rest parameters of the intervention conducted by this group during the 8 weeks (three times per week). WBV was provided with a Galileo 900 (Novotec Medical GmbH, Pforzheim, Germany). This device makes fast oscillating movements around the sagittal.

Placebo group protocol: As commented above, participants of PG believed that they were receiving a WBV below the vibration threshold (unable to detect). For this purpose, the Galileo Fitness platform was connected through wiring to a screen where the participant had the values of vibration duration and frequency amplitude, but it really was a software that controlled two speakers placed inside the vibration platform. Previous studies have performed a similar procedure [33,34].

All participants, irrespectively of their group allocation, were required to: (1) stand on the platform on their feet at an equal and standardized distance from the middle axis (4 mm of vibration amplitude); (2) keep their eyes fixed forward with the back straight; (3) maintain a knee flexion at 45°; and (4) get on the platform without shoes. In order to ensure the security of the protocol, all participants were encouraged to follow the instructions. Moreover, a physical therapist supervised the intervention in order to correct the posture when appropriate and to control the whole-body vibration protocol for each training day. Additionally, the intervention protocol was progressive in both vibration time and intensity as shown in Table 1.

### 2.4. Outcomes

Lipid profile, HbA1c, fasting blood glucose, body composition, and blood pressure: The lipid profile (total cholesterol, high-density lipoprotein -HDL- and low-density lipoprotein -LDL-) and the HbA1c were obtained through a blood sample in the morning after an overnight fast. Moreover, participants were measured and weighed using the Tanita Body Composition Analyzer BC-418 MA. The blood pressure was measured using an aneroid sphygmomanometer.

Foot health status: The Foot Health Status Questionnaire (FHSQ) was used in the present study. It is composed of three sections. The first section includes 13 questions and evaluates four dimensions (Foot Pain, Foot Function, Shoe, and General Foot Health). The second section includes 20 questions and evaluates the four dimensions left (General Health, Physical Activity, Social Capacity, and Vigour). Each question is valued following a Likert-type scale and the scoring goes from 0–100, where 0 is the worst foot health status and 100 is the best possible foot health status. Lastly, the third section refers to the socio-demographic values of the sample. The validated version adapted to Spanish was employed [35].

Health-related quality of life: The 15-D Health Questionnaire [36] was used in the present study. This questionnaire evaluates 15 Dimensions (Mobility, Vision, Hearing, Breathing, Sleep, Eating, Speech, Elimination, Habitual Activities, Mental Function, Discomfort and Symptoms, Depression, Anxiety, Vitality, and Sexual Activity). Each dimension has five possible answers from less to more impaired. The total score represents the person’s health status (1—full HRQoL, 0—death).

Chair-stand test: This test evaluated lower-limb strength by counting the number of times the participant is able to stand up from a sitting position (i.e., until reaching complete knee extension) and then sit down as many times as possible in 30 s [37]. This test has shown a high reliability in this population [38].

Timed-up go (TUG): The TUG (3m) was performed two times, with a 5-min rest in between. The first repetition was for familiarisation and the second was the test measure. All participants performed a light warm-up which included walking and joint mobility for 5 min. Reliability analysis for this test in the DM population has been previously published [38,39].

### 2.5. Statistical Analysis

The SPSS statistical package (version 20.0; SPSS, Inc., Chicago, IL, USA) was used to analyse the data. Non-parametric tests were conducted based on the results of Shapiro-Wilk and Kolmogorov-Smirnov tests.

Data from all 90 initial participants were used to conduct the intention-to-treat analysis by multiple imputation (MI) of missing values following the Sterne, et al. [40] guidelines. Our missing data were classified as missing at random. The SPSS software was used to perform MI of data.

Mann-Whitney U test was employed to study the differences at baseline in the physical function, self-perceived health foot status, HbA1c, fasting blood glucose or body composition variables among others. Moreover, the difference between post and pre was calculated for each variable. These differences were used to study between-group effects of the intervention by the Mann-Whitney U test in the different variables.

Moreover, Wilcoxon signed-rank test was used to explore the within groups effects for each variable (WBV and placebo group separately). Additionally, effect sizes were calculated for the non-parametric tests through r, which is classified as follows: 0.5 is a large effect, 0.3 is a medium effect, and 0.1 is a small effect [41,42].

## 3. Results

**Figure 1:**
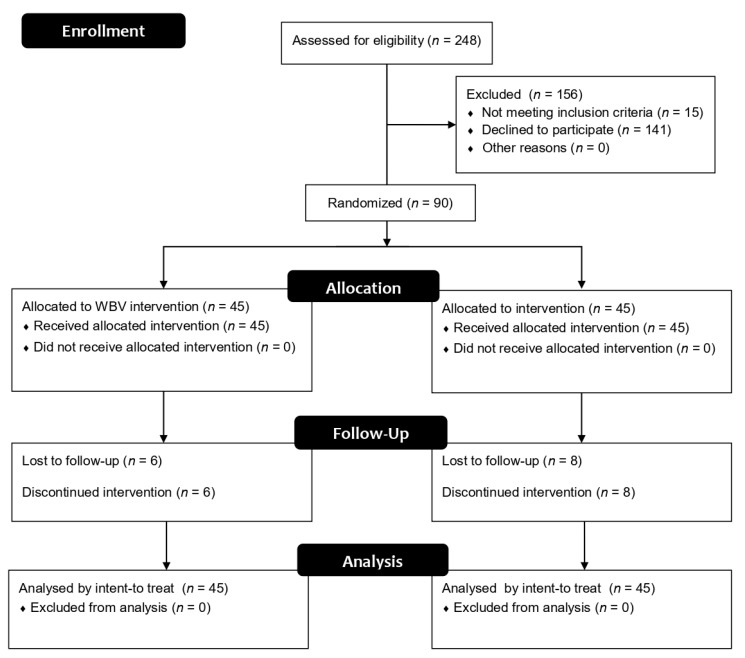
Flow diagram of participants.

The flow diagram of the participants is depicted in Figure 1. A total of 248 people with T2DM were assessed for eligibility. However, 156 were excluded. Therefore, 90 adults with T2DM were randomized into two groups: WBV (*n* = 45) and PG (*n* = 45). Six and eight participants of the WBV and the PG respectively did not attend the final evaluation. In this regard, intent to treat analysis were applied to this data.

The main characteristics of the included participants are shown in Table 2. No significant differences were observed at baseline in any of the variables.

Table 3 shows the effects of the WBV intervention in the body composition and HbA1c. In the WBV group, within-group results showed significant increases in free fat mass (*p*-value = 0.006), total body water (*p*-value = 0.009), HDL (*p*-value = 0.036) and a significant decrease in BMI (*p*-value = 0.003), fat mass (*p*-value = 0.003), total cholesterol (*p*-value = 0.005), systolic blood pressure (*p*-value = 0.002), fasting blood glucose (*p*-value = 0.022) and HbA1c (*p*-value = 0.001). The PG showed significant decreases of weight (*p*-value = 0.004), BMI (*p*-value = 0.025), systolic blood pressure (*p*-value < 0.001), fasting blood glucose (*p*-value < 0.001), and HbA1c (*p*-value = 0.004). Between-group comparisons showed that WBV significantly reduced the fat mass (*p*-value = 0.049).

Table 4 shows the effects of the intervention in FHSQ. The WBV and PG showed significant increases in the shoe FHSQ item (*p*-value = 0.015) and vigour FHSQ item (*p*-value = 0.034) respectively. Mann-Whitney U did not show any significant effect.

Table 5 shows the effects of the intervention in the health-related quality of life, TUG and stand test. Within-group analyses showed that both WBV and PG enhanced in these variables. However, between-groups analyses did not find any significant effects of the WBV.

## 4. Discussion

Our study showed that 8 weeks of WBV training had a significant effect on the body composition, reducing the fat mass of people with T2DM. However, significant effects on mobility, HbA1c, blood glucose, blood pressure, lipid profile, or health-related quality of life were not found. Nevertheless, within-groups improvements in WBV in physical fitness, body composition, foot health status, systolic blood pressure, HbA1c, lipid profile or fasting blood glucose showed that WBV training could be included as part of therapeutic intervention for people with T2DM. Importantly, side effects were not reported for any of the participants at 12.5–18.5 Hz.

In line with our result, previous studies have shown significant effects of WBV on the body composition of older adults with T2DM. In this regard, Sanudo, Alfonso-Rosa, Del Pozo-Cruz, Del Pozo-Cruz, Galiano, and Figueroa [29] found a 7.2% body fat mass reduction after 12 weeks of WBV training. In this intervention, authors followed a similar vibration procedure (12–16 Hz and 30–60 s of duration), however, they combined dynamic and static exercises. In other populations, such as post-menopausal women, these changes have been also observed when WBV was combined with resistance training [43]. Since people with T2DM are usually obese and have an excess of abdominal fat, which increases the risk of developing both metabolic and cardiovascular diseases, it is relevant that only with passive WBV changes in body fat mass could be obtained [44]. Regarding HbA1c levels, a significant decrease in both groups was found. However, no significant effect of the WBV was detected. Similarly, effects on blood glucose, lipid profile or blood pressure were not found. However, the WBV training group significantly enhanced these parameters (blood glucose, lipid profile, and blood pressure). These results are in line with previous studies which did not find a significant effect of WBV on HbA1c [25,26]. However, there are also studies which have observed a positive effect of WBV on HbA1c, fasting blood glucose or cholesterol [27,28]. Therefore, further studies are needed to clarify the effects on these variables.

The effects of WBV training on quality of life and foot health in people with T2DM have been poorly studied. Regarding quality of life, previous studies showed that WBV could improve the quality of life in older populations [45] and other chronic conditions [46,47]. In this regard, a previous study of 6 weeks of WBV improved all domains (pain, general and mental health, vitality, physical, emotional, and social functioning) of the SF-36 quality of life questionnaire in people with diabetic neuropathy. In our study, effects of 8-weeks WBV training on quality of life were not found. Nevertheless, within-groups improvements in health-related quality of life were found in both EG and placebo group. Similar to these results, effects on FHSQ were not found. However, to the best of our knowledge, this is the first study reporting the effects of FHSQ after WBV training. Therefore, further studies are needed to confirm this hypothesis.

Results of physical function indicated that both groups (WBV and PG) obtained within-group improvements in the chair stand test and TUG. However, significant effects of the WBV training were not found. Nevertheless, previous studies have reported positive effects of WBV (with or without complementary exercises) on the physical function of people with T2DM [48]. Moreover, this is in line with previous results that showed similar improvements in lower-limb muscle strength [49,50]. Regarding the TUG, this test can assess a person’s mobility since it requires both static and dynamic balance [51]. A previous study found a 32% improvement in the TUG after WBV training in people with T2DM [52]. However, there are other studies that did not observe any effect of this intervention on the TUG [28,53].

In most of the studied variables, an improvement could be observed in both the EG and placebo group after 8 weeks of intervention. It could hypothetically be explained by two reasons. First, people with T2DM have high levels of physical inactivity [5]. Simply by their participation in the intervention, participants of both groups have to move three times per week to the primary care facilities in order to perform the intervention. Therefore, the participants were more active than before the intervention. Second, even the placebo group experienced the positive social effect of being enrolled in a physical activity intervention. This could be the reason why self-perceived quality of life and foot health improved in the placebo group after 8 weeks.

The current study has some limitations that should be taken into account. Firstly, the relatively small sample size (*N* = 90) did not allow to create different group of patients with different diabetic conditions (i.e., with diabetic neuropathy). Consequently, the subgroups would have had a sample size too small to make comparisons with enough statistical power strength. In the same line, we would have also found the same problem in a study that aimed to compare the effects of different vibration frequency protocols. Therefore, future studies should investigate both the effects of WBV training in different diabetic conditions and the dose-response of different WBV protocols. Lastly, this randomized controlled trial included a placebo group that did not statistically differ in any of the variables at baseline when compared with the WBV group. This group followed the same protocol to WBV but with an infra-threshold vibration (simulated with two speakers placed under the vibration platform). Therefore, they only had to perform the same protocol isometric contraction on the platform. Taking into account the physical inactivity level of people with T2DM [5], this could explain the improvements on physical conditions that were found in the PG.

## 5. Conclusions

WBV reduced the fat mass of people with T2DM. However, significant effects were not found in HbA1c, physical function, foot health, and quality of life. Further studies are needed to clarify the effects of WBV in people with T2DM.

## Figures and Tables

**Table 1:** Whole-body vibration procedures for each week of the intervention.

Week	Number of Series per Session	Duration of the Serie (seconds)	Vibration Frequency (Hertzios)	Rest between Series (seconds)	Intervention Duration per Week (seconds)
1	8	30	12.5	30	720
2	5	60	13.5	30	900
3	6	60	14.5	30	1080
4	7	60	15.5	30	1260
5	8	60	16.5	30	1440
6	9	60	17.5	30	1620
7	9	60	18.5	30	1620
8	9	60	18.5	30	1620

**Table 2:** Baseline characteristics of the study participants.

Variables	All (*n* = 90)	WBV Group (*n* = 45)	Placebo Group (*n* = 45)	*p*-Value
Weight (kg)	80.63 (6.19)	81.78 (18.04)	79.48 (14.22)	0.926
BMI (kg/m^2^)	29.65 (4.39)	30.37 (5.28)	28.93 (3.17)	0.298
Fat Free Mass (%)	32.98 (7.49)	33.50 (7.73)	32.45 (7.30)	0.583
HbA1c (%)	6.78 (1.02)	6.78 (1.15)	6.79 (0.89)	0.572
Total cholesterol	172.74 (35.59)	168.37 (34.66)	177.12 (36.34)	0.081
Systolic Blood Pressure	140.28 (22.52)	142.63 (21.41)	137.93 (23.57)	0.165
Blood Glucose	131.29 (30.62)	132.50 (33.54)	130.07 (27.71)	0.870
GFH of the FHSQ	57.96 (25.08)	57.72 (25.43)	58.20 (25.01)	0.760
Timed-Up and Go (s)	8.12 (2.01)	8.29 (2.28)	7.96 (1.71)	0.696
Chair Stand Test	11.51 (2.24)	11.91 (2.24)	11.11 (2.21)	0.063
15D HRQoL Questionnaire	0.89 (0.09)	0.90 (0.09)	0.89 (0.10)	0.777

WBV—Whole-body vibration; BMI—Body Mass Index; HbA1c—Glycosylated haemoglobin; GFH—General Foot Health; FHSQ—Foot Health Status Questionnaire; HRQoL—Health-Related Quality of Life; kg—kilogrames; m—meters; cm—centimeters; s—seconds.

**Table 3:** Intent-to-treat of 8-weeks WBV training in the body composition, blood pressure, lipid profile, blood glucose, and HbA1c.

Body Composition Variables				Within Group Comparison			Between Group Comparison		
	Groups	Pre	Post	Z	*p*-Value *	Effect Size	Z	*p*-Value *	Effect Size
Weight (kg)	WBV	81.78 (18.04)	81.35 (17.90)	−2.25	0.056	−0.33	−0.92	0.404	−0.10
	Placebo	79.48 (14.22)	78.43 (14.00)	−2.99	0.004 *	−0.45			
BMI (kg/m^2^)	WBV	30.37 (5.28)	28.43 (2.95)	−2.98	0.005 *	−0.44	−0.77	0.445	−0.08
	Placebo	28.93 (3.17)	30.21 (5.24)	−2.32	0.025 *	−0.34			
Free fat mass (%)	WBV	66.11 (8.81)	67.27 (7.62)	−2.98	0.006 *	−0.44	−1.79	0.104	−0.19
	Placebo	67.57 (7.30)	67.53 (7.24)	−0.32	0.757	−0.48			
Fat mass (%)	WBV	33.50 (7.73)	32.83 (7.77)	−3.04	0.003 *	−0.45	−2.132	0.049 *	−0.22
	Placebo	32.45 (7.30)	32.60 (7.20)	−0.36	0.740	−0.05			
Total body water (%)	WBV	48.70 (5.64)	49.25 (5.73)	−2.91	0.009 *	−0.43	−2.039	0.070	−0.21
	Placebo	49.47 (5.33)	49.37 (5.31)	−0.24	0.811	−0.03			
Basal metabolic rate (kcal)	WBV	1606.23 (339.80)	1610.21 (339.50)	−0.85	0.418	−0.13	−1.597	0.133	−0.17
	Placebo	1589.96 (319.06)	1578.59 (325.11)	−1.49	0.148	−0.22			
HbA1c (%)	WBV	6.78 (1.16)	6.54 (0.97)	−3.19	0.001 *	−0.47	−0.307	0.765	−0.03
	Placebo	6.79 (0.89)	6.60 (0.91)	−2.94	0.004 *	−0.44			
Total cholesterol (mg/dL)	WBV	168.37 (34.66)	158.01 (25.97)	−3.00	0.005 *	−0.44	−0.697	0.554	−0.07
	Placebo	177.12 (36.34)	170.17 (28.05)	−2.08	0.080	−0.31			
HDL (mg/dL)	WBV	49.22 (12.18)	50.18 (10.44)	−2.38	0.036 *	−0.35	−0.659	0.548	−0.07
	Placebo	52.45 (15.00)	54.15 (14.52)	−1.75	0.133	−0.26			
LDL (mg/dL)	WBV	91.47 (31.14)	83.32 (23.58)	−2.54	0.018 *	−0.38	−0.463	0.652	0.05
	Placebo	99.93 (32.47)	98.17 (42.76)	−1.77	0.164	−0.26			
Systolic blood pressure (mmHg)	WBV	142.63 (21.41)	127.57 (27.58)	−3.87	0.002 *	−0.58	−0.289	0.800	0.03
	Placebo	137.93 (23.57)	125.44 (25.09)	−3.51	<0.001 *	−0.52			
Fasting blood glucose (mg/dL)	WBV	132.50 (33.54)	122.15 (31.67)	−2.41	0.022 *	−0.36	−1.514	0.176	0.16
	Placebo	130.07 (27.71)	115.27 (26.72)	−4.35	<0.001 *	−0.65			

Note: * *p*-value < 0.05; BMI—Body Mass Index; HbA1c—Glycosylated haemoglobin; HDL—High-Density lipoprotein; LDL—Low-Density lipoprotein.

**Table 4:** Intent-to-treat of 8-weeks WBV training in the Foot Health Status Questionnaire (FHSQ) dimensions in people with T2DM.

Variables				Within Group Comparison			Between Group Comparison		
	Groups	Pre	Post	Z	*p*-Value *	Effect Size	Z	*p*-Value *	Effect Size
Foot Pain	WBV	87.99 (21.94)	93.64 (16.02)	−1.78	0.118	−0.26	−0.661	0.551	−0.07
	Placebo	89.50 (19.85)	94.00 (14.94)	−1.63	0.131	−0.24			
Foot function	WBV	94.86 (15.38)	97.03 (10.27)	−1.02	0.353	−0.15	−0.856	0.457	−0.09
	Placebo	93.64 (17.30)	95.74 (12.52)	−1.50	0.317	−0.22			
Shoe	WBV	79.44 (36.26)	90.75 (25.22)	−2.61	0.015^*^	−0.39	−0.815	0.485	−0.08
	Placebo	71.39 (39.98)	79.79 (35.25)	−1.79	0.074	−0.27			
General foot health	WBV	57.72 (25.43)	62.97 (30.20)	−1.45	0.187	−0.22	−0.408	0.703	−0.04
	Placebo	58.20 (25.01)	62.19 (21.54)	−1.21	0.233	−0.18			
General Health	WBV	69.11 (22.85)	71.68 (24.15)	−1.13	0.285	−0.17	−0.981	0.331	−0.10
	Placebo	64.36 (23.86)	69.92 (22.32)	−1.69	0.107	−0.25			
Physical Activity	WBV	85.43 (16.59)	85.64 (14.75)	−0.45	0.658	−0.07	−1.205	0.258	−0.13
	Placebo	75.34 (25.80)	79.94 (20.81)	−1.42	0.190	−0.21			
Social capacity	WBV	94.17 (16.12)	96.31 (16.66)	−0.76	0.469	−0.11	−0.894	0.394	−0.09
	Placebo	87.99 (24.18)	93.66 (20.07)	−1.98	0.053	−0.29			
Vigour	WBV	72.78 (19.86)	74.65 (17.71)	−0.94	0.367	−0.14	−1.297	0.220	−0.14
	Placebo	61.84 (26.26)	69.11 (23.06)	−2.28	0.034 *	−0.34			

* *p*-value < 0.05.

**Table 5:** Intent-to-treat of 8-weeks WBV training in the TUG, chair stand test and 15-D HRQoL Questionnaire.

Physical Fitness and Quality of Life				Within Group Comparison			Between Group Comparison		
	Groups	Pre	Post	Z	*p*-Value *	Effect Size	Z	*p*-Value *	Effect Size
TUG (s)	WBV	8.29 (2.28)	7.42 (1.72)	−4.24	<0.001 *	−0.63	−1.718	0.095	−0.18
	Placebo	7.96 (1.71)	7.49 (1.41)	−2.82	0.006 *	−0.42			
Chair-stand test (rep)	WBV	11.91 (2.24)	12.96 (2.24)	−4.03	<0.001 *	−0.60	−1.316	0.256	−0.14
	Placebo	11.11 (2.21)	11.70 (2.31)	−2.96	0.005 *	−0.44			
15D HRQoL Questionnaire	WBV	0.90 (0.09)	0.94 (0.07)	−4.28	<0.001 *	−0.64	−0.723	0.526	−0.08
	Placebo	0.89 (0.10)	0.93 (0.06)	−4.52	<0.001 *	−0.67			

* *p*-value < 0.05; HRQoL—Health-Related Quality of Life; rep—repetitions; TUG—Timed-up and go test.
